# Aptamer‐Based Delivery Systems for VEGF and NGF Modulation in Ocular Therapies

**DOI:** 10.1002/adhm.202505182

**Published:** 2026-04-22

**Authors:** Nadine Best, Gizem Karabiyik, Gerd Geerling, Sarah Barbara Zwingelberg, Gregor Lang, Martin Humenik

**Affiliations:** ^1^ Department of Biomaterials University of Bayreuth Prof.‐Rüdiger‐Bormann‐Str. 1 Bayreuth Germany; ^2^ Department for Functional Materials in Medicine and Dentistry University Hospital of Würzburg Pleicherwall 2 Würzburg Germany; ^3^ Department of Ophthalmology University of Düsseldorf Moorenstr. 5 Düsseldorf Germany

**Keywords:** aptamers, corneal neovascularization, growth factors therapies, implants, ophthalmic drug delivery

## Abstract

Many diseases that threaten vision originate from imbalances in the production or signaling of vascular endothelial growth factor (VEGF) and nerve growth factor (NGF). While VEGF inhibitors can suppress pathological blood vessel growth and NGF therapies can promote tissue regeneration, treatments employing the growth factors are often invasive, inefficient, or not suitable for long‐term use. For example, the use of NGF‐containing eye drops requires high drug loads and frequent administration, thereby increasing the potential for side effects and high costs. Based on a critical overview of currently used experimental and clinical approaches for the delivery or inhibition of growth factors, we highlight aptamers as promising yet underexplored therapeutic tools. Aptamers combine high target specificity, tunable structure and affinity, stability, low immunogenicity, and scalable chemical synthesis. These properties allow aptamers to be readily incorporated into biomaterial carriers, such as hydrogels, implants, or contact lenses, to improve the controlled binding of growth factors for precise modulation of their levels in ocular tissues via release or sequestration strategies. Leveraging these advantages in future developments could facilitate the aptamer‐functionalized platforms to overcome the limitations of current therapies and advance more sustainable, targeted, and patient‐oriented treatment strategies in ophthalmology.

## Graphical abstract

Aptamer‐based delivery systems targeting NGF and VEGF hold significant potential for precise and sustained treatment of complex diseases affecting both the anterior and posterior segments of the eye. This review critically summarizes current strategies for NGF and VEGF delivery as well as VEGF sequestration, providing a clinical perspective on how aptamer‐functionalized platforms may advance next‐generation ophthalmic therapies.

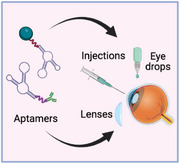

## 1 Introduction

The eye consists of two major sections. The anterior part includes the transparent cornea, the pigmented iris, the conjunctiva, the lens, and the ciliary body, whereas the posterior section includes the retina, the vitreous humor, the sclera, the choroid, the optic disc, and the optic nerve. In case of ophthalmic pathologies, drug delivery systems play a crucial role in addressing various ocular diseases by delivering drugs locally, thereby increasing treatment efficacy [1, 2, 3, 4].

Significant advancements have been achieved in recent years in effective targeting, ocular bioavailability, therapeutic duration, patient compliance, and minimizing side effects. However, the static, dynamic, and metabolic barriers of the eye collectively restrict drug access to target tissues, thereby reducing therapeutic efficacy [5, 6, 7]. To overcome these challenges, various strategies have been developed, including novel formulations and physical force‐based techniques [6]. Nanocarrier systems such as liposomes and nanoparticles have been engineered to bypass ocular barriers, enhance drug permeation, improve targeting, and increase bioavailability [8, 9, 10, 11, 12, 13]. In addition, in vitro and ex vivo models were developed for evaluating the potential of drug delivery systems to cross the eye barriers [14]. In addition to the physical circumvention of barriers using nanocarriers, the development of targeted molecular ligands has become a key focus of modern ocular therapies. Due to their biocompatibility and modularity, aptamers offer a highly tunable platform for the precise treatment of diseases in both the anterior and posterior segments.

### 1.1 Aptamers – Nucleic Acid‐Based Targeting Molecules

Aptamers (Latin: *aptus* (fitting); Greek: *meros* (particle)) are short single‐stranded oligonucleotide sequences with a molecular weight of 6000–12,000 Da, which bind a plethora of targets, such as metal ions, small organic molecules, proteins, viruses, or even cells, with a high affinity [15, 16].

**FIGURE 1:**
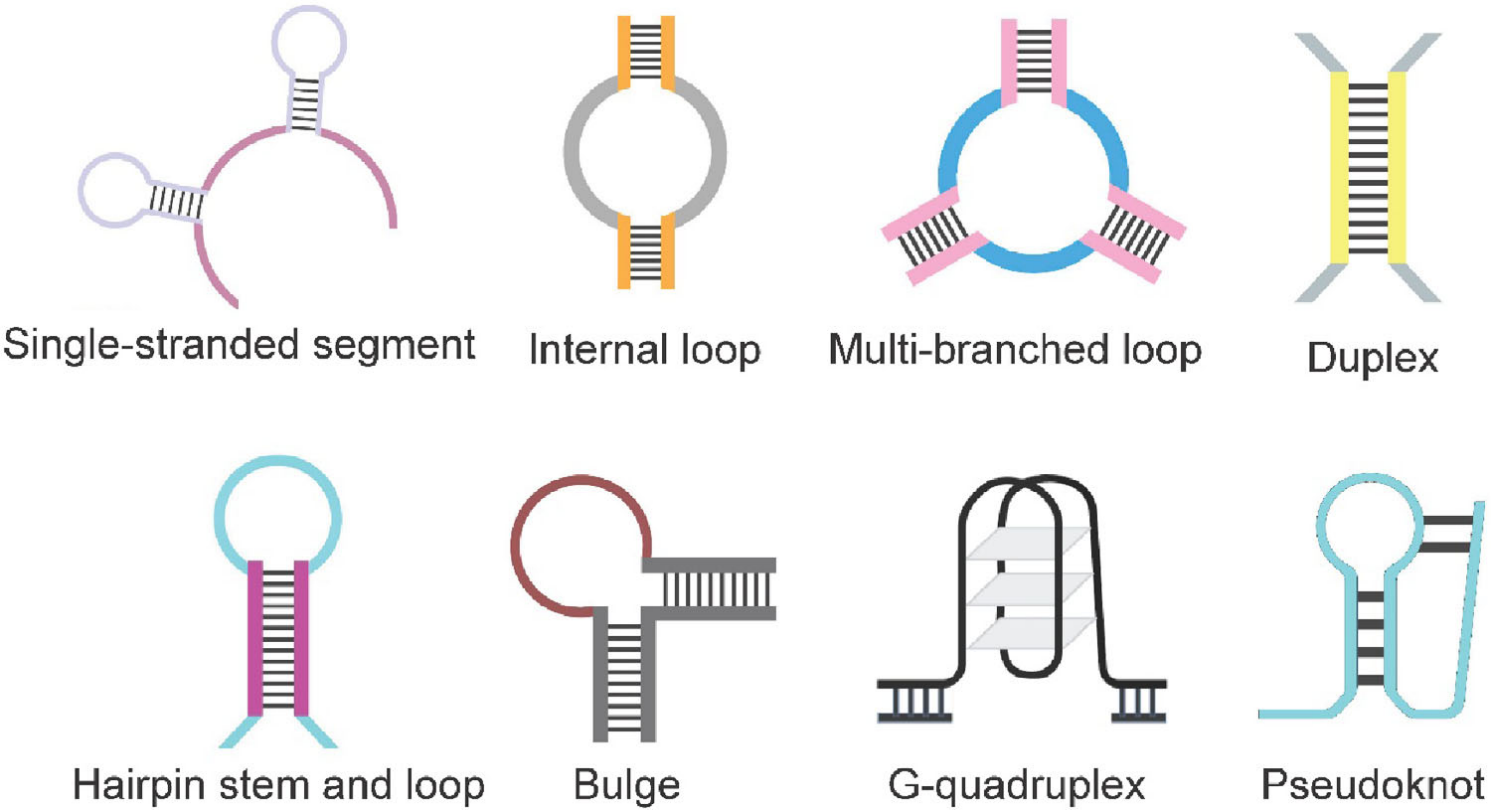
The secondary structure motifs of aptamers include loops, duplexes, bulges, hairpins, as well as more complex structures, such as G‐quadruplex and pseudoknot. Created by Biorender.

The binding specificity of aptamers is typically defined by their distinct secondary structures, e.g., loops [17], duplexes [18], bulges [17], and hairpins [19, 20] (Figure 1), resulting in spatially oriented interactions of the nucleobases and/or sugar‐phosphate backbone with the target [21]. Besides these, non‐Watson‐Crick base pairing can further lead to complex tertiary structure patterns [22] like the G‐quadruplex formed by guanine‐rich sequences, or G‐triplex structures formed by guanine triads [23, 24]. The G‐quadruplex motifs are typically stabilized with cations, e.g., Na^+^ or K^+^. Compared to a duplex‐forming motif, the G‐quadruplex includes a doubled negative charge density, which supports the binding of the often positively charged targets, such as protein domains [25, 26]. In addition, diverse structural motifs of an aptamer might interact with each other and form more complicated 3D architectures known as pseudoknots (Figure 1) [27, 28]. These folding regimes promote the formation of unique 3D‐molecular architectures, enabling the high aptamer binding specificity [29].

Aptamers are based on ribonucleic (RNA) or deoxyribonucleic (DNA) sequences identified in a process called Systematic Evolution of Ligands through Exponential Enrichment (SELEX), comprising screening a chemically synthesized randomized library (10^13^–10^16^ sequences) in selection rounds, resulting in high‐affinity candidate sequences with K_d_ values often in the nanomolar range [30, 31, 32]. Within the SELEX process, positive and negative controls as well as stringent conditions are utilized to identify highly specific aptamer binders. The customization within the screening process allows the identification of a matching sequence for a specific application [33]. Moreover, non‐SELEX processes have been developed to speed up the sequence selection. These are based on screening methods, omitting the amplification step at the end and involving, e.g., molecular filtration, magnetic beads, or centrifugation forces to sort out unbound sequences, hence shortening the selection process down to only one or a few rounds [34, 35]. High specificity and affinity binding of aptamers toward their targets resemble the principal antibodies; thus, aptamers are also referred to as nucleic acid antibodies [36].

### 1.2 Aptamers vs. Antibodies as Molecular Therapeutics

Monoclonal antibodies (mAbs) have become indispensable tools in diagnostics, therapeutics, and research nowadays. The traditional production technology using hybridoma cells, i.e., a fusion of murine B cells with myeloma cells, presents challenges, including hybridoma instability and the generation of murine antibodies, which can elicit human anti‐mouse antibody responses. Advanced techniques have been developed to address these limitations, such as humanized mice, to express fully human mAbs following immunization. Alternatively, antigen‐specific B‐cell cloning allows for producing antibodies of interest and shows stable, long‐term expression. Additionally, in vitro engineering offers precise control over mAb design with desired characteristics, e.g., grafting murine variable domains onto human constant domains yields chimeric antibodies with minimized immunogenicity while retaining antigen‐binding specificity [37, 38]. However, producing and purifying antibodies remain laborious and expensive. Their delicate tertiary structure limits their environmental stability. Moreover, antibodies’ typically high affinities to antigens exclude applications requiring dynamic release kinetics. Another limitation of antibodies is their potential to interact with the receptors on cell surfaces or with the immune system [37, 39].

Aptamers can overcome some limitations of antibodies for specific applications. They are produced cost‐efficiently and consistently through solid‐phase synthesis, possess low immunogenicity, are significantly smaller (6–12 kDa) and simpler‐structured, providing more environmental stability. In addition, the SELEX process offers a customized development of aptamers with suitable binding affinities to match application purposes [39, 40, 41, 42]. Despite the many benefits, a few challenges have also been identified. These include instability against nucleases, which, however, can be effectively addressed with chemical modifications, [43, 44] as discussed in Section 6.

Although the antibodies are more established in clinical studies and commercial biomedical use, aptamers are more often included in a material science context [45, 46, 47]. Through the continued translation of material concepts into therapeutic applications, the clinical use of aptamers is expected to expand significantly in the near future. In particular, their potential in ophthalmology is highly promising. When combined with appropriate biomaterials or advanced drug delivery platforms, aptamers could help overcome challenges associated with ocular complexity, biological barriers, and target specificity. Such strategies may substantially advance ocular therapeutics, especially when directed toward suitable therapeutic targets, such as growth factors (GFs) that play crucial roles in maintaining eye homeostasis and regulating healing processes.

## 2 Biological Role of Growth Factors in Ophthalmology

To clarify the challenges encountered in the treatment of ocular disorders, we first briefly describe ocular anatomy and the associated biological barriers, which are essential for advancing drug administration–based therapies. In the second part, we focus on GFs and their roles in eye homeostasis and diseases, to better understand their potential as promising therapeutic targets and agents.

**FIGURE 2:**
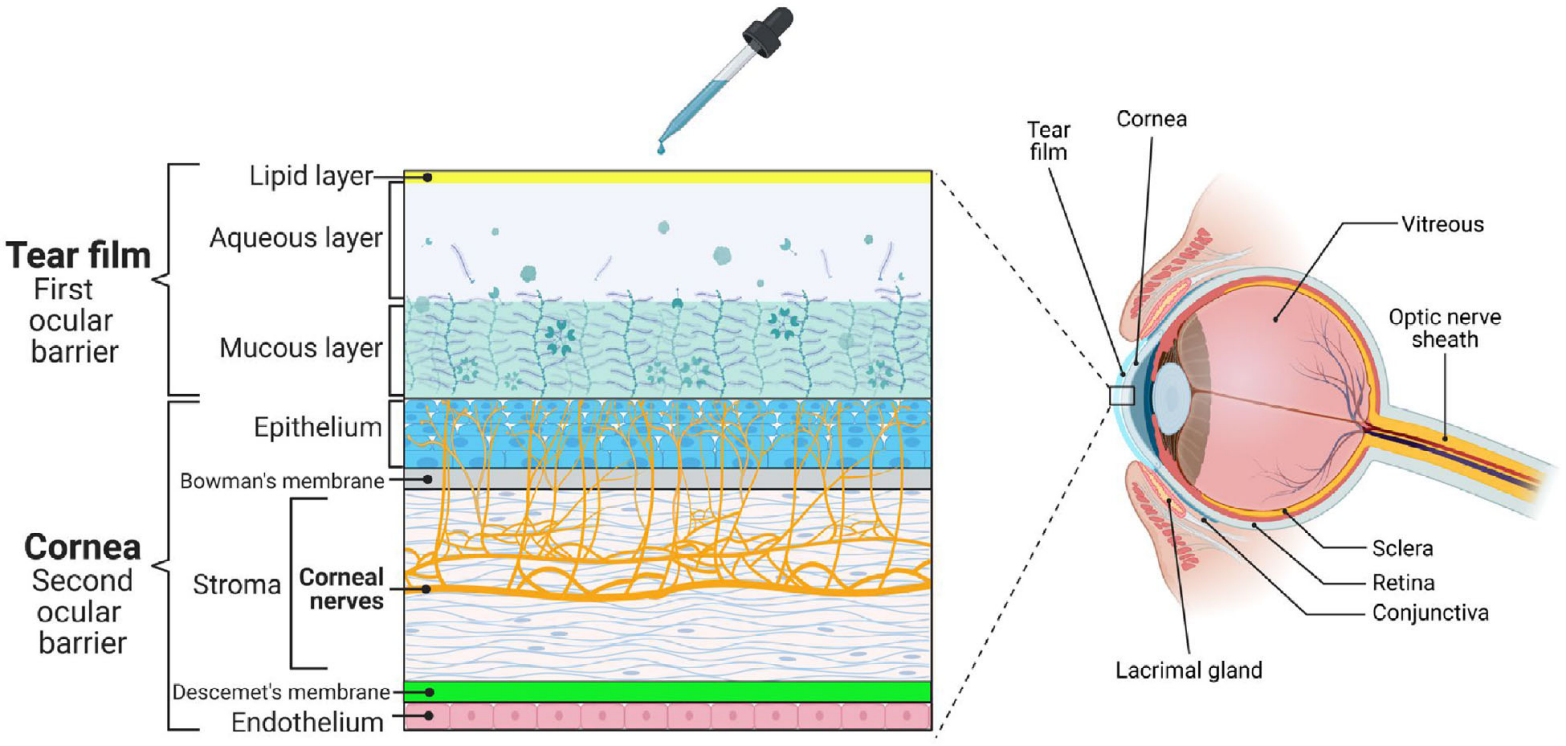
Eye frontiers. Ocular tear film and cornea are the main ophthalmic barriers of the eye for the use of topical drugs, like eye drops. Components of these barriers are represented. Corneal nerves are shown in orange. Designed with Biorender.

As shown in Figure 1, the tear film is composed of aqueous and mucin layers. It contains lipids and enzymes, such as lysozyme, which have antimicrobial properties, and represents the first barrier. The second barrier is the cornea, composed of five layers, as indicated in Figure 1. The lipophilic epithelium permits the passage of small hydrophobic compounds, whereas the hydrophilic stroma enables the passage of hydrophilic compounds. Meanwhile, the corneal endothelium selectively allows the transport of hydrophilic drugs and large molecules into the aqueous humor. In contrast, the conjunctiva is more permeable to hydrophilic drugs and allows drug delivery to the iris and ciliary body. The blood‐aqueous barrier in the anterior part and the blood‐retinal barrier in the posterior segment, which can be seen in Figure 2, control the entry of substances into the bloodstream [1, 48, 49].

The corneal nerves play a crucial role in ocular surface homeostasis and visual function [50, 51] as well as in the production of various neurotransmitters and neuropeptides for corneal maintenance and repair [52, 53]. Dysfunction of corneal nerves leads to a range of ocular conditions, including dry eye disease, neurotrophic keratitis (NK), and neuropathic pain [54, 55, 56]. Conversely, corneal pathologies can themselves induce nerve abnormalities [53, 54, 55]. Therefore, understanding the intricate crosstalk is principal for developing targeted therapeutic strategies that preserve corneal health and visual function [50, 57].

Considering the complexity of the eye structure, numerous GFs regulate physiological processes in ocular tissues, maintaining visual function, as well as being involved in wound healing and disease mechanisms. Notably, NGF, VEGF, epidermal growth factor (EGF), fibroblast growth factor (FGF), platelet‐derived growth factor (PDGF), and transforming growth factor‐beta (TGF‐β) are expressed across ocular structures and contribute to different mechanisms, which are summarized in Table 1 [58, 59].

**TABLE 1:** Key GFs in Ophthalmology.

Growth factor	Biological function	Main ocular roles
NGF	Neuroprotection, nerve regeneration	Regulating immune response, regulating corneal and conjunctival epithelial cell proliferation, and healing
VEGF	Angiogenesis	Retinal neovascularization (endothelial cell proliferation and the formation of tubular structures)
EGF	Epithelial cell proliferation	Corneal wound healing, growth, and migration of lens epithelial, production of tears, tear neuroregulatory pathway
FGF	Cell proliferation and differentiation, angiogenesis	Development and differentiation of retina and lens, corneal repair
PDGF	Fibrosis, cell migration	Wound remodeling, vitreoretinal interface, proliferation, survival, and migration of retinal cells
TGF‐β	Immune modulation, fibrosis	Fibrosis, inflammation, deposition of extracellular matrix, and corneal wound healing.

In general, we focus in the review on NGF and VEGF due to their central roles in two major pathological pathways in ocular biology: angiogenesis and neurodegeneration, as discussed in the next sections. Therefore, understanding the interplay of NGF and VEGF, as well as their receptor interactions, may facilitate the development of novel therapeutic approaches [60], particularly regarding their potential as targets for aptamer‐based therapies.

### 2.1 Nerve Growth Factor

NGF is a soluble protein belonging to the neurotrophic factor family, whose importance is underlined by the widespread expression in both the anterior and the posterior chambers [61, 62]. NGF is critical for maintaining neuronal functions, on one hand promoting growth, differentiation, and regeneration of nerve cells, and on the other hand impacting regulatory pathways for the synthesis of neurotransmitters and neuropeptides. In addition, NGF also affects the immune and endocrine systems, interacting with non‐neuronal cells, such as epithelial cells, glial cells, keratinocytes, fibroblasts, immune cells, neuroendocrine cells, etc. Therefore, NGF is established for the treatment of central and peripheral nervous system disorders as well as ocular diseases, e.g., corneal and skin ulcers, retinitis pigmentosa, glaucoma, optic nerve degeneration, ocular inflammation, etc. [63, 64, 65, 66].

**FIGURE 3:**
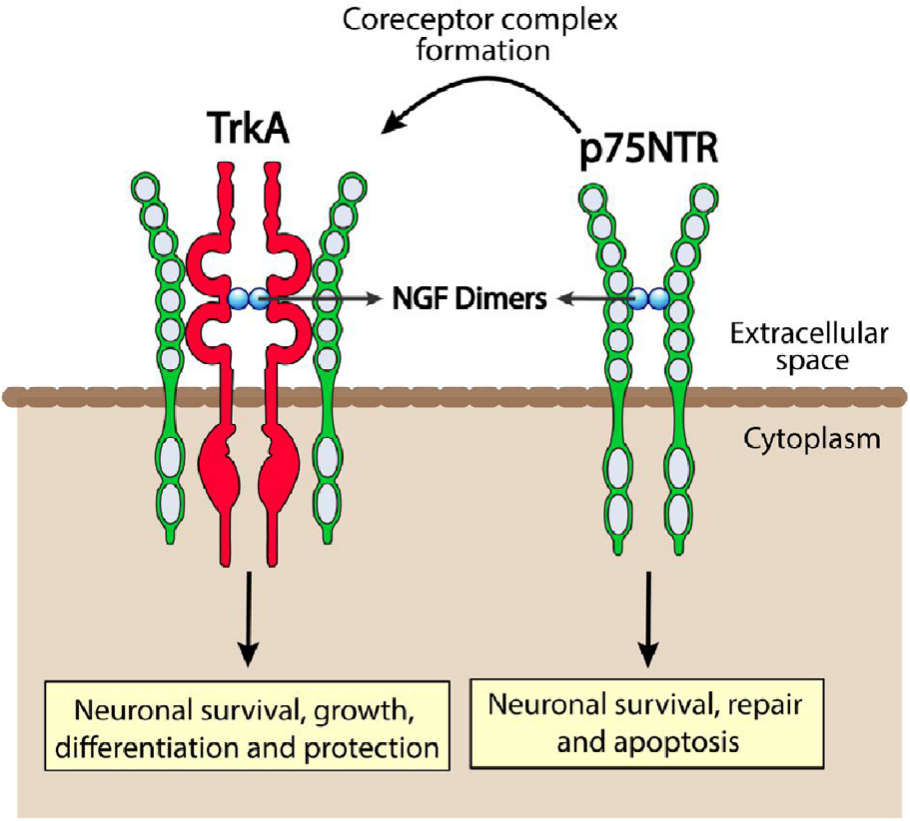
Summary of ocular NGF signaling pathways activated by binding the TrkA or p75NTR receptors, forming a coreceptor complex. NGF dimer binding to the TrkA receptor regulates neuronal proliferation, growth, differentiation, survival, and protection via activation of different pathways. NGF dimer binding to the p75NTR receptor mediates neuronal survival, apoptosis, or neural repair. Designed with Adobe Illustrator.

There are two NGF receptors, as illustrated in Figure 3: Tropomyosin receptor kinase A (TrkA) and p75 neurotrophin (NT) receptor (p75NTR), which are present in the cornea, conjunctiva, and ciliary body. NGF dimers specifically bind to TrkA receptors with a high affinity, thus activating intracellular signaling pathways promoting cell survival, proliferation, growth, and differentiation of target neurons [64, 67, 68]. p75NTR is a low‐affinity receptor that plays a role in neurotrophic signaling pathways like cell survival, apoptosis, or neural repair. Furthermore, the TrkA and p75NTR receptors interact with each other and form a coreceptor complex to reinforce the role of NGF on both neuronal and non‐neuronal cells [64, 67]. NGF also affects the expression of microRNAs (miRNAs), short, non‐coding RNAs that act in the post‐transcriptional regulation of genes. Recent studies revealed that NGF could be a potent signaling protein for treating corneal epithelial diseases by targeting miRNAs [69, 70, 71].

**A) NGF in Anterior Segment**

NGF is abundantly produced in the anterior parts, such as corneal epithelium, stroma, conjunctival epithelium, and the tear film, highlighting its importance for the corneal surface [72, 73]. NGF preserves corneal nerve activity, which is typically compromised in neurotrophic diseases, such as NK [70, 72, 73]. Many corneal pathologies damage the epithelium [74], and ocular surface trauma can cause severe pain. NGF promotes epithelial healing, nerve regeneration, and pain relief following injury [52]. Ocular surface defects, inflammation, and allergies also induce the expression of NGF and its receptors at sites of damage [75, 76, 77]. Overall, NGF plays a key role in corneal physiology, homeostasis, wound healing, and pathology, highlighting its therapeutic potential in a wide range of corneal disorders [78, 79].

**B) NGF in the Posterior Segment**

**FIGURE 4:**
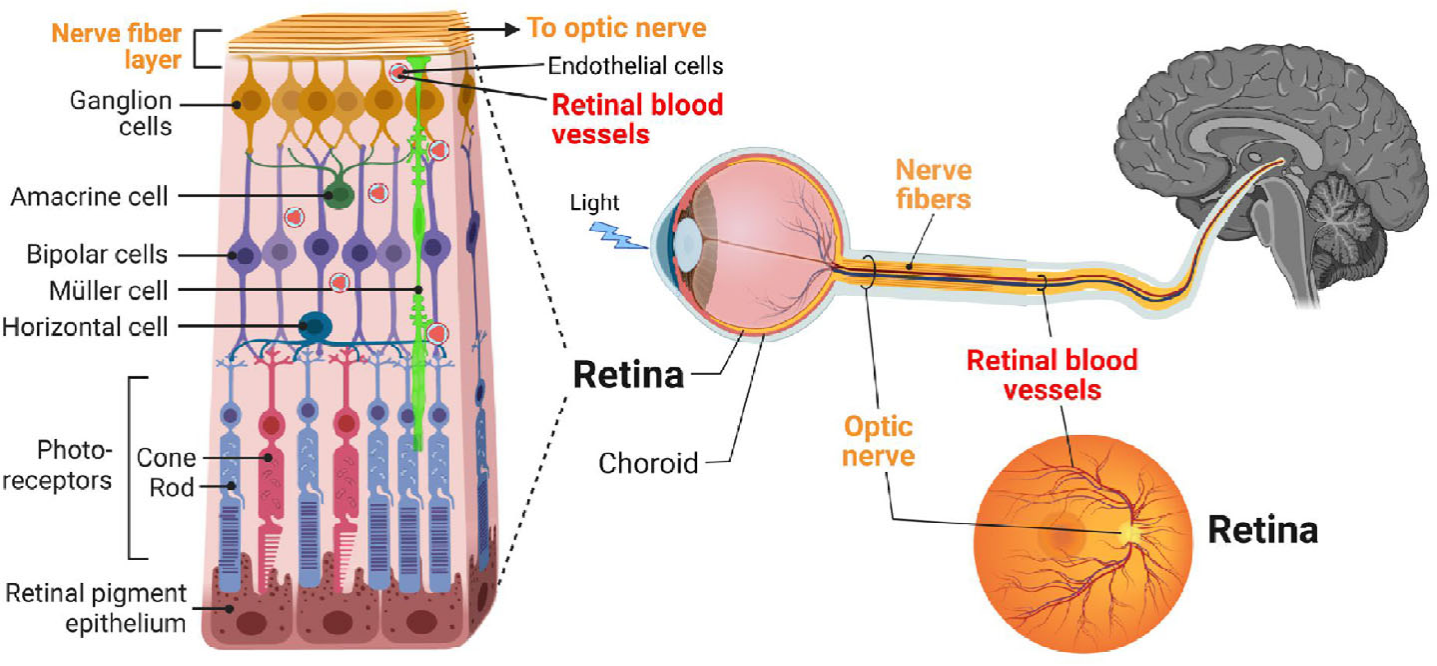
Eye backdoor. Layers and cell types in the human retina have complex nervous and vascular connections between the ocular system and the CNS. Retinal nerves are shown in orange, and retinal vessels are demonstrated in red. Designed with Biorender.

In the posterior segment, the retina – connecting the ocular system and the central nervous system (CNS) – is composed of different layers as illustrated in Figure 4. Especially, Müller cells, pigment epithelial cells, and retinal ganglion cells (RGCs) are known to produce NGF [49, 80, 81]. In case of axon damage, such as optic nerve injury or glaucoma, the nerve connections are disrupted, causing RGC dysfunction and death. Since their axons have no self‐healing ability, this may potentially result in irreversible vision loss. NGF, however, may restore RGC survival and function, supporting axonal growth and regeneration [82].

### 2.2 Vascular Endothelial Growth Factor

**FIGURE 5:**
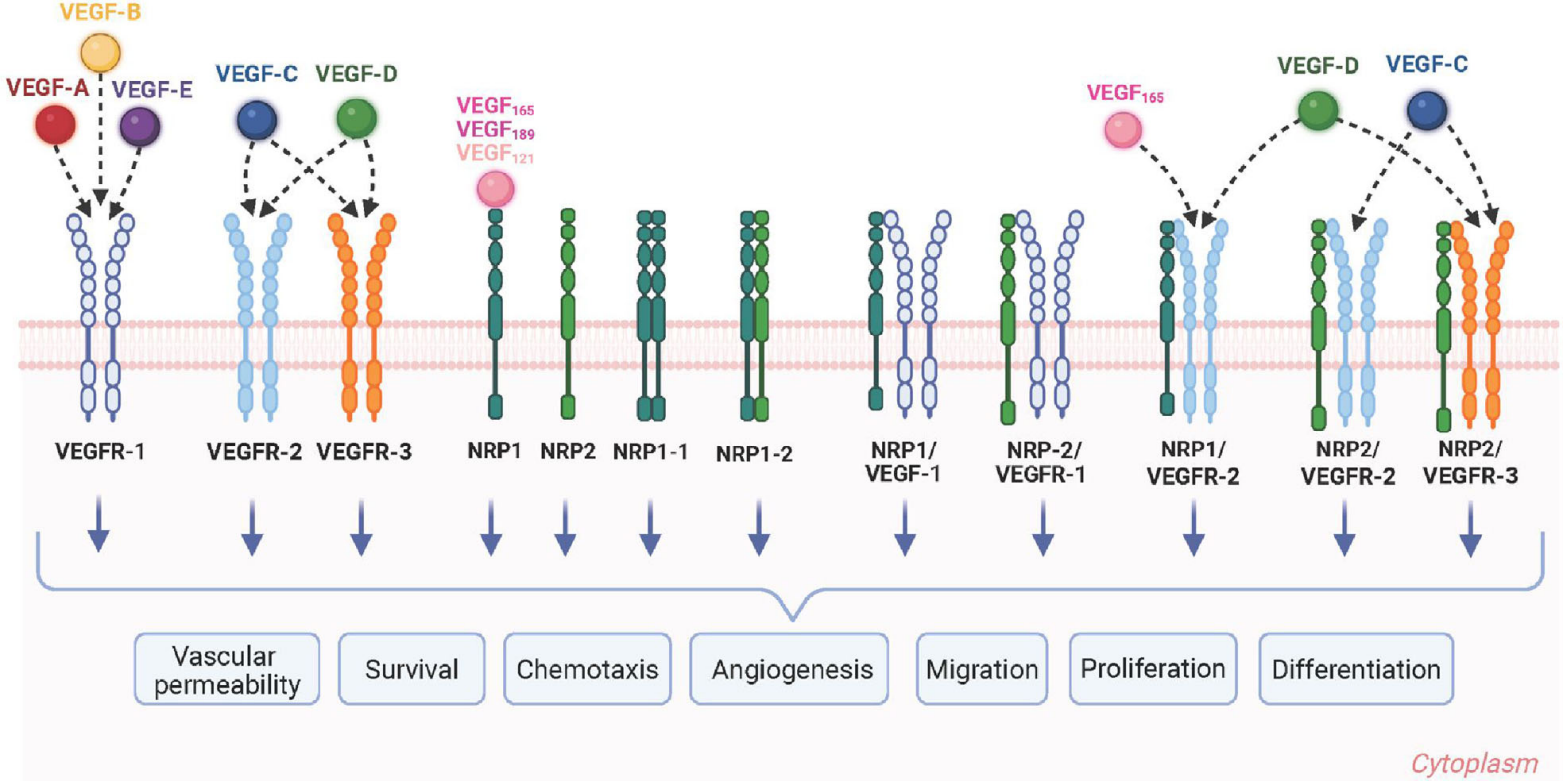
Overview of VEGF receptors, including neuropilins NRP. Specific types of VEGF bind to distinct receptors or receptor complexes, triggering diverse vascular signaling pathways. Designed by Biorender.

VEGFs are a family of growth factors promoting vasculogenesis during the formation of the primary capillary plexus, as well as angiogenesis – building new capillaries from existing blood vessels. However, VEGFs are also involved in pathological effects, e.g., causing intraocular neovascular disorders or supporting tumor growth. The biological effects of VEGF are mediated through its binding to receptors (VEGFRs) – a group of cognate protein tyrosine kinase receptors (Figure 5) [83, 84].

VEGF subtypes are classified based on their binding to VEGFRs, with VEGF‐A being the most prominent due to its roles in endothelial cell proliferation, tissue homeostasis, and tumor progression [85, 86, 87]. Alternative mRNA splicing generates multiple VEGF‐A isoforms among which VEGF_165_ is particularly important [87, 88, 89, 90]. Secreted VEGFs signal through VEGFR‐1 and VEGFR‐2, together with neuropilin co‐receptors (NRP‐1 and NRP‐2), which modulate angiogenic signaling in ocular vascular systems, as summarized in Figure 5. Notably, VEGF_165_ preferentially binds NRP‐1 and promotes receptor complex formation, thereby driving key vascular signaling pathways [89, 91, 92, 93, 94]. Overall interactions of NRPs and VEGFs lead to specific and complex signaling pathways in ocular systems involving further growth factors, which have been reviewed in detail by Raimondi et al. [94].

Ocular structures of the anterior and posterior segments interact with VEGF in diverse ways. Hence, the role of VEGF in both segments is reviewed in the next two sections, separately.

**A) VEGF in Anterior Segment**

Most anterior structures are supplied by blood, excluding the avascular, transparent lens and cornea [95, 96, 97]. The avascularity of the cornea is counterintuitively maintained by bound soluble *fms*‐like tyrosine kinase‐1 (sVEGFR‐1) binding, and hence inhibiting the VEGF‐A [98]. sVEGFR‐1 is an alternatively spliced isoform of VEGFR‐1 that lacks tyrosine kinase and transmembrane domains. Interestingly, sVEGFR‐1 has been found in the cornea, but not in the neighboring conjunctiva that secretes the membrane‐bound variant VEGFR‐1 [99]. The balance between pro‐angiogenic and anti‐angiogenic factors at the corneal surface is maintained and distributed by the tear film [100]. In addition to the sVEGFR‐1/VEGF complex, other anti‐angiogenic mediators, such as pigment epithelium–derived factor (PEDF), counteract the angiogenic activity of VEGF [101, 102]. The alymphatic nature of the cornea, a feature closely associated with its immune privilege, is similarly preserved by soluble VEGFR‐2, which sequesters VEGF‐C and VEGF‐D, thereby inhibiting lymphangiogenesis [103, 104, 105].

This equilibrium of angiogenic and anti‐angiogenic factors can be disturbed by pathological incidences, leading to neovascularization and pathological corneal remodeling [98]. This process contributes to vision impairment and blindness, which collectively affect over 10 million individuals worldwide [95, 106]. The primary therapeutic intervention in such cases is human donor tissue transplantation; however, the risk of graft failure or immune rejection remains a significant challenge [107]. The presence of pre‐existing lymphatic vessels within the corneal limbus, adjacent to the transplantation site, has been implicated in promoting immune‐mediated graft rejection. Understanding the intricate interplay between corneal neovascularization, lymphangiogenesis, and immune surveillance is therefore essential for the development of novel therapeutic strategies aimed at improving graft survival and minimizing transplant rejection [105, 108].

**B) VEGF in Posterior Segment**

In contrast to the cornea, the neural retina in adult humans possesses a proper blood supply, as seen in Figure 2, arising from the retinal and the choroidal vessels [109]. The vascularization of the retina is primarily based on angiogenesis triggered by VEGF [89, 110, 111]. The VEGF secretion in the ocular system occurs via endothelial cells, retinal pigmented epithelium, ganglion cells, Müller cells, and astrocytes, triggered by diverse conditions [89, 112, 113]. Under hypoxia, astrocytes and Müller cells were found to secrete larger amounts of VEGF [89]. Severe eye injury will also promote vascularization via VEGF secretion [112, 113]. However, the angiogenesis process requires a balance between the formation and destruction of blood vessels to prevent pathological vascularization and diseases, such as tumor growth, proliferative retinopathies, AMD, DR, and central vein occlusion [112, 114, 115, 116].

In summary, both NGF and VEGF play central roles in maintaining ocular homeostasis, regeneration, and healing processes; therefore, they represent meaningful therapeutic targets for aptamer‐based delivery strategies.

### 2.3 Aptamers Targeting NGF and VEGF

The next section will review currently identified NGF and VEGF‐targeting aptamers, highlighting their potential impact in these fields.

**A) NGF‐Targeting Aptamers**

NGF‐targeting aptamers are rarely described within the current literature. Binkley et al. evolved RNA‐based aptamers, one of which featured a complex secondary structure, moderate affinity (*K_d_* = 100 nM), and, notably, binding stability under high salt conditions. The second RNA aptamer lacked a defined structure, and its binding to NGF was sensitive to elevated salt concentrations [117]. Variation in resistance to high salt concentrations highlights the importance of optimizing screening conditions. Nakamura et al. screened for DNA‐based and RNA‐based sequences (> 45 bases) with high affinity to NGF [118]. In 2015, Jarvis et al. introduced a novel class of modified aptamers showing slow off‐rates, known as SOMAmers. The sequences of SOMAmers are shorter than those in Nakamura et al. and incorporate hydrophobic nucleotide 5‐(N‐benzyl carboxamide)‐2'‐deoxyuridine, which increases binding specificity by adopting a non‐helical, compact, S‐shaped structure utilizing extensive hydrophobic stacking interactions and a novel, intercalating, zipper‐like motif [119].

Notably, none of these aptamers have yet been used in ophthalmology. The current underdevelopment of NGF‐targeting aptamers indicates that this is an unexplored area of research with enormous potential for NGF‐related therapies.

**B) VEGF‐Targeting Aptamers**

In contrast to NGF‐targeting aptamers, a broad range of VEGF‐targeting aptamers has been described. Notably, pegaptanib (*Ma* *cugen*) became the first aptamer approved for ophthalmologic therapy [120, 121]. The delivery of pegaptanib occurred utilizing PLGA microspheres, and the release profile was reported in a sustained manner over 20 days [121]. Pegaptanib was tested in many different clinical studies, summarized by Bege et al., to treat AMD, DR, DME, and corneal neovascularization [122]. However, aptamer‐based therapies were subsequently outcompeted by antibody‐based anti‐VEGF treatments, and for nearly two decades, pegaptanib remained the only FDA‐approved therapeutic aptamer in ophthalmology until the recent approval of avacincaptad pegol (*Izelvay*) for geographic atrophy secondary to AMD [122].

In current developments, the bispecific aptamer VED‐LYTAC, which targets both VEGF and the mannose‐6‐phosphate receptor, represents a promising example of multifunctional aptamer engineering, enabling VEGF sequestration and lysosome‐mediated degradation [123]. VED‐LYTAC was introduced for the management of angiogenic retinal disorders and represents a novel route compared to common strategies, such as anti‐VEGF antibodies, to inhibit the interaction of VEGF with VEGFR. In comparison to the mAb bevacizumab, VED‐LYTAC showed a superior efficacy after one injection, likely due to higher in vivo stability and penetration depth [123]. In this context, Nonaka et al. identified the V7t1 aptamer, which binds both VEGF_165_ and VEGF_121_ with high affinity (*K_d_* of 1.4 nM and 1.1 nM, respectively). V7t1 targets the receptor‐binding domain (RBD) of VEGF, in contrast to pegaptanib, which binds the heparin‐binding domain (HBD). To enhance sensitivity and specificity toward VEGF_165_, which contains both domains, V7t1 was fused with del5‐1, an aptamer targeting the HBD, which significantly improved the binding with a *K_d_* of 470 pM [124]. Referring to the V7t1 aptamer, Kurth et al. modified the V7t1 aptamer with fluorescein to develop a target‐induced dissociation assay, which has been applied in relation to AMD‐related bioanalytics [125, 126]. Beyond the molecular engineering, aptamer conjugation to delivery systems, such as carbon dots, has been explored as a hybrid nanocarrier for AMD and DR treatments. These hybrids achieve therapeutic levels via topical administration, exhibit no toxicity in both in vitro and in vivo murine models, and allow noninvasive monitoring of intraocular concentrations through the inherent fluorescence of the C‐dots. Importantly, the system effectively inhibits VEGF‐induced angiogenesis in choroidal vessels, with efficacy comparable to the commercially available anti‐VEGF drugs bevacizumab and aflibercept [127]. V7t1, VED‐LYTAC, and aptamer–carbon dot hybrids illustrate the potential of aptamer engineering to enhance therapeutic precision and address current limitations in AMD treatment. These examples also emphasize the value of structural insights in aptamer design and highlight the promise of multivalent constructs for targeted therapeutic applications. Although VEGF‐binding aptamers are more widely developed in biomedical materials and ocular disorder treatments than NGF‐targeting aptamers, their clinical application remains limited, representing a promising research venue.

The following sections provide an overview of established systems to explore the potential for developing novel drug delivery systems using NGF‐ and VEGF‐targeting aptamers. The therapeutic concepts center on delivering NGF‐ or VEGF‐inactivating agents. However, targeted VEGF delivery to the eye remains unexplored.

## 3 Delivery Systems Impacting NGF and VEGF Levels in Ocular Therapies

**FIGURE 6:**
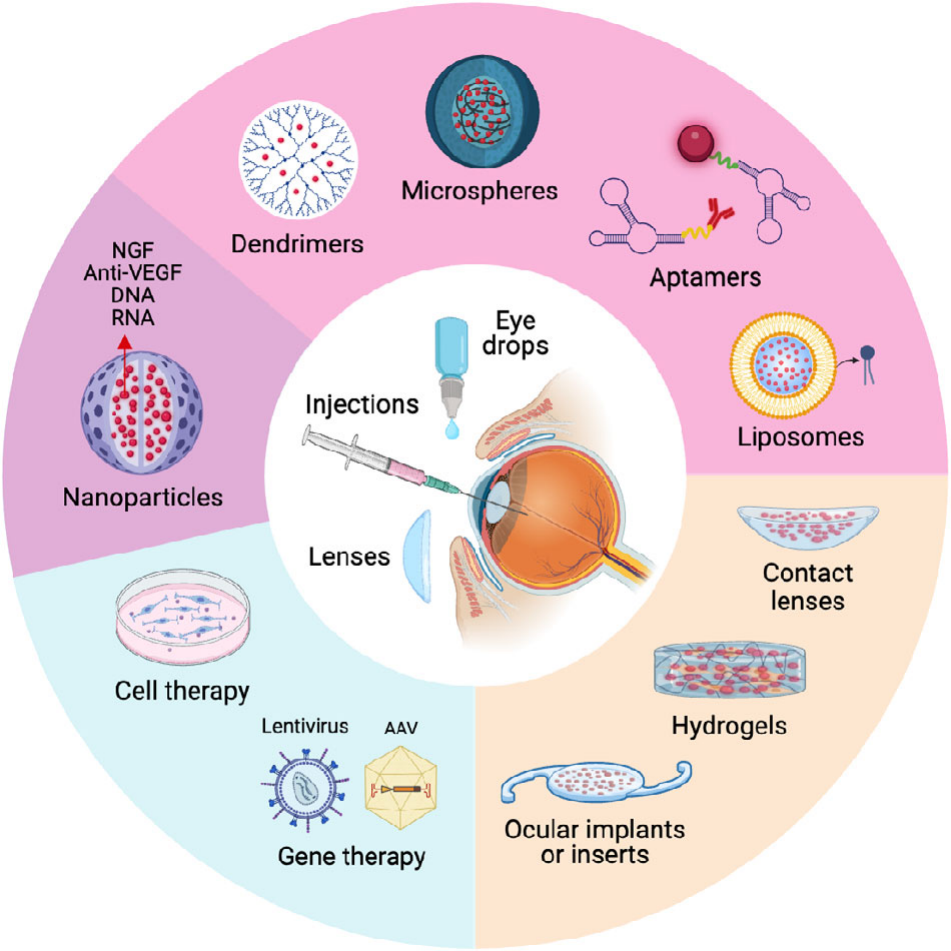
Delivery approaches involving NGF and/or VEGF for the treatment of ocular disorders. Color‐coded regions represent three classes of carrier systems: nanocarrier‐based systems (pink), including nanoparticles, microspheres, dendrimers, liposomes, and aptamers; gene and cell therapy strategies (blue), including lentiviral/AAV‐based gene delivery, *ex vivo* cell therapeutic approaches, and nanoparticle‐based DNA and RNA delivery; implantable and hydrogel‐based platforms (orange), such as contact lenses, hydrogels, and ocular implants or inserts. Designed with Biorender.

This section examines delivery systems for neurotrophic NGF and angiogenic VEGF in ophthalmology in both anterior and posterior segments, which pathologies commonly lead to vision loss. From the available administration strategies (Figure 6), we reviewed those material platforms that are suitable for integrating aptamers in the further development of ocular therapies.

### 3.1 Administration and Delivery of NGF

Numerous studies have used exogenous administration of natural, synthetic, or recombinant protein forms of NGF for ocular therapies. In these forms, amino acids in the NGF protein may be modified, substituted, or deleted in comparison to the natural sequence. Conversely, small NGF peptide derivatives have been developed, which may provide better bioavailability and stability compared to full‐length NGF [128, 129, 130, 131].

NGF can be delivered via several routes, including oral administration, intracerebroventricular or intravenous injections, nasal spray, or direct ocular applications [64, 75, 131]. In addition to the peptide forms, NGF can be intrinsically delivered as mRNA or DNA to the cells to express NGF [128, 129, 130, 131]. Systemic routes generally require high doses to achieve therapeutic effects in the eye, which increases the risk of side effects and toxicity in non‐target organs [64, 131]. Therefore, localized methods, such as topical, intraocular (intracameral or intravitreal), or periocular administration, have been the focus in order to minimize systemic exposure and enhance ocular bioavailability [64].

**A) Topical Administration**

Using eye drops for NGF delivery is straightforward in applicability. In vivo studies have demonstrated that topical NGF administration to the eye can prevent cellular loss, promote axonal growth, and support regeneration in both anterior and posterior segments [132, 133, 134]. Furthermore, the NGF eye drops trigger the reproduction and differentiation of epithelial and endothelial cells, as well as fibroblasts, and initiate their migration from the edges toward the damaged center [135, 136]. Hence, the topical use of NGF in different ocular diseases, such as dry eye [137], neurotrophic keratitis [138], peripheral ulcerative keratopathy [139], glaucoma [140], corneal neurotrophic ulcers [132], retinal neurodegeneration, etc., opened new perspectives in the past three decades. A prominent example is cenegermin (*Oxervate*), a commercially available eye drop containing rhNGF, which promotes corneal epithelial healing and nerve regeneration [68, 138, 141]. Despite its therapeutic potential, cenegermin presents several practical challenges. In general, topical drug administration is highly inefficient. The average volume of a single eye drop is ranging 25–56 µL, which is about two to five times greater than the holding capacity of the conjunctival sac (≈10 µL). Hence, 50–80% of the drug immediately overflows the eye, further enhanced due to the blinking reflex [142]. For instance, the recommended regimen for cenegermin requires administration six times daily for eight weeks, which may impose a considerable burden on patients. Furthermore, the high cost (approximately $2000 per vial) has raised cost‐effectiveness concerns and influenced reimbursement recommendations in certain countries due to the predicted budget impact.

Considering the limitations outlined above, such as poor corneal retention, rapid drug overflow, high dosing frequency, and considerable treatment cost, there is a significant demand for the development of a new cost‐effective NGF delivery system [12, 64, 68, 143], which could be realized through aptamer‐functionalized contact lenses as an alternative NGF delivery platform to control the GF‐release (see also Section C).

**B) Intraocular Injections**

Intraocular injection is used as an alternative to topical eye drops to administer drugs, especially for disorders related to the posterior segment. However, NGF has not been studied as extensively in posterior segment disorders, unlike VEGF or VEGF‐inhibiting agents [144].

In early studies, the neuroprotective ability of NGF using the intraocular injection effectively improved the viability and function of the RGCs and optic nerve fibers in animal models, decelerating retinal degeneration and enhancing the thickness of the retinal outer nuclear layer, where the nuclei of photoreceptors are located [145, 146, 147, 148]. No translation into clinical application has been demonstrated so far. Intraocular injection of NTs in ophthalmology faces some challenges, especially short half‐life, leading to multiple injections, which, however, may increase the risk of cataract, retinal ischemia, and endophthalmitis in addition to the arising pain and discomfort of patients. Long‐term treatment may also cause receptor downregulation, risk of infection, increased intraocular pressure, or other unknown effects, underscoring the need for alternative, more efficient delivery methods [149]. The short half‐life, receptor downregulation risk, and complication burden associated with repeated intraocular NGF injections highlight the need for sustained‐release systems with precisely tunable release kinetics (see Section 6.3). Aptamer‐functionalized injectable hydrogels represent a promising approach in this context: by immobilizing low‐affinity NGF‐binding aptamers within a hydrogel matrix (see also Section 6), the system can act as a molecular reservoir, releasing NGF in a sustained manner that avoids the concentration peaks responsible for receptor desensitization.

**C) Contact Lenses**

Contact lenses have also gained attention as an option for ocular drug delivery. Therapeutic agents are loaded by simply immersing the lenses in concentrated drug solutions, while the drugs are then gradually released via passive diffusion. Soft contact lenses made of hydrogels, such as gelatin [150], collagen [151], silicone [150], can absorb drugs due to their porous structures. In the context of contact lens‐based drug delivery systems, water content, oxygen permeability, and thickness of the hydrogel, as well as hydrophobicity, molecular weight, concentration, and retention time of the drug are critical aspects. Contact lenses can improve the bioavailability of the drug in comparison to traditional eye drops. However, burst release regimes are common under such passive release systems [152, 153, 154, 155], leading to fast diffusion through the corneal epithelium into the anterior chamber, which limits their therapeutic efficacy.

Moreover, distinct pharmacological agents necessitate a tailored lens carrier material to allow drug‐specific loading and release profiles. To the best of our knowledge, there are currently no studies, either clinical or preclinical, reporting the use of contact lenses as carriers for NGF delivery in ocular diseases. However, preclinical studies have demonstrated the use of hydrogel‐based contact lenses for the delivery of EGFs to promote ocular wound healing [155]. In addition, chitosan microspheres containing NGF have been integrated into collagen–chitosan scaffolds to repair peripheral nerve injuries [156]. These findings indicate that such strategies could potentially be translated to contact lenses for NGF‐based ocular applications. Unlike current passive drug‐loaded lenses, lenses functionalized with NGF‐binding aptamers (as mentioned already in Section A), could exploit the reversible, affinity‐based interaction between aptamers and growth factors to buffer the release profile, sustaining therapeutic concentrations over extended wear periods. The affinity‐tunable nature of aptamers further allows the release rate to be matched to the corneal epithelial renewal cycle. In this context, the clinical experience with cenegermin establishes both the therapeutic proof‐of‐concept for topical NGF delivery and a clear performance benchmark that aptamer‐functionalized platforms must meet or exceed. Section 6 discusses further capabilities of aptamer integration into biomaterial platforms for GF delivery.

**D) Nanocarriers for NGF Delivery**

The latest advancements in NGF delivery have been driven by innovative nanocarrier systems to increase therapeutic efficacy in neurological and ocular disorders [157]. Exemplarily, Giannaccini et al. conjugated NGF with iron oxide nanoparticles. Histological data demonstrated that the conjugated proteins totally prevented RGC loss, in sharp contrast to the free proteins, which had no effect. Overall, the data suggested that the conjugation of NGF to the nanoparticles improves the NGF stability and activity [149]. In a recent study, polyacrylamide nanoparticles conjugated with NGF and peanut agglutinin were used. In vitro and in vivo experiments in the zebrafish model showed that the peanut agglutinin facilitated the targeting of the ocular posterior segment and increased the retention time of the conjugated NGF [158]. Iron oxide nanoparticles covalently modified with NGF were used in a magnetic field to direct the delivery. The advantages of this approach are drug stability, biocompatibility, and efficient penetration into tissues and cells. The limitations are the use of external magnets and the decreasing intensity of the magnetic field in deeper tissues [159]. However, none of the nanocarrier systems have been tested in a clinical setting yet, and several obstacles remain. The production and resuspension of nanoparticles often require processes like stirring, heating, sonication, and the use of organic solvents, which could have a negative impact on NGF's structure and bioactivity [66]. Moreover, some inorganic and synthetic polymer nanoparticle systems may face bio‐incompatibilities in terms of degradation and increased immune responses in prolonged periods after application. These limitations should be considered when developing nanocarriers for delivery systems, since the therapeutic integrity and biocompatibility of biomolecules such as NGF are crucial for their efficacy and success [66, 160].

In general, depending on the target site, NGF delivery strategies may be limited by the blood‐ocular barriers (blood‐vitreous, blood‐aqueous, and blood‐retina), NGF's high molecular weight, hydrophilicity, negative charge, low stability, short retention time, and off‐target effects. Therefore, the feasibility of NGF‐based deliveries in ophthalmology requires the use of cutting‐edge systems that maintain persistent release, maximize bioavailability, and minimize systemic effects to achieve targeted and efficient treatment outcomes [64, 152, 161, 162]. When aptamers are used as the functional surface element and the available immobilization chemistries are employed under mild aqueous conditions (as reviewed in Section 6.1), aptamer‐functionalized nanoparticles can improve the non‐specific surface coatings (e.g., PEGylation) that are currently used to prolong nanoparticle circulation. At the same time, they offer a more targeted and tunable approach for a binding GF of interest.

### 3.2 Administration and Delivery of VEGF

VEGF is central to signaling pathways for ocular vascularization. Various VEGF administration strategies have already been reviewed by Beheshtizadeh et al. [163], which are used regenerative medicine by promoting the vascularization in bone, cardiac, and skin tissues [164, 165]. To the best of our knowledge, no applications have been reported for ocular regeneration. Such approaches could encounter significant challenges, such as the need for precise control of dosage and the target site. Inappropriate VEGF levels could lead to adverse effects, including hemorrhaging, excessive or insufficient angiogenesis, negative feedback inhibition, tumor formation, or downregulation of VEGF signaling receptors.

Here, we summarize recent hydrogel‐based delivery platforms that incorporated VEGF for tissue engineering and regenerative applications, which could potentially be adapted to integrate aptamers for use in ocular therapies. These systems can be formulated as printable or injectable constructs, enabling the controlled and targeted release of VEGF and supporting their strategic use in ocular therapies.

Hydrogel‐based systems include modified biocompatible biopolymers, such as chitosan, alginate, and fibrin [166]. Fibrin is involved in natural wound healing, and the hydrogels made thereof showed a sustained VEGF release upon cell infiltration and successive matrix degradation [163, 167]. Furthermore, alginate, an algae‐based and FDA‐approved polysaccharide, received attention due to its biocompatibility, biodegradability, and easy processing [168, 169]. Alginate hydrogels reinforced with electrospun poly(l‐lactide‐co‐ε‐caprolactone) (PLCL)/gelatin methacryloyl (GelMA) coaxial nanofibers have been loaded with VEGF. In vitro studies showed a sustained release of bioactive VEGF for at least 14 days, making the modified alginate system a promising candidate for improving the beta cell survival [170]. Another polysaccharide system based on methacrylated hyaluronic acid (HAMA) microspheres was loaded with VEGF, and the system promoted new blood vessels and endometrial regeneration of a thin endometrium in vivo [171].

Synthetic polymers have been reported for VEGF delivery as well. A branched, 4‐arm polyethylene glycol (PEG) was chemically cross‐linked to form hydrogels or hydrogel‐based microspheres, demonstrating local perfusion and GF release leading to functional vascularization [172, 173]. Furthermore, a PEG‐hydrogel‐based and poly(lactic‐co‐glycolic acid) (PLGA)‐mPEG block copolymer system was reported that utilized norbornene and dithiol click‐chemistry to immobilize peptide ligand obtained from VEGFR‐2 that could effectively bind and release VEGF‐A in a slow and sustainable manner [172, 174]. PLGA microspheres show generally efficient encapsulation and slow‐release profiles for VEGF due to naturally occurring hydrolysis of the polymer scaffolds [175, 176]. Other injectable hydrogel‐based systems employed chitosan nanofibrous microspheres in a PLGA‐PEG‐PLGA hydrogel doped with VEGF. The rapid release of VEGF promoted the swift initiation of angiogenesis [177, 178, 179, 180]. General trend shows the development of more complex microsphere‐loaded hydrogels combining natural and synthetic biopolymers for controlled GF release.

Another strategy to overcome the limitations of GF release from hydrogels features three‐dimensional (3D) printing, which offers the advantage of cost and time‐efficient fabrication of complex geometric shapes with various inks [181, 182]. Besides, the variable hydrogel materials, more complex 3D‐printed systems integrate VEGF‐binding and delivering molecules, whereby the precise deposition of material and the possibility of creating complex geometries offer new perspectives in controlled delivery of VEGF [183]. Strategies for 3D‐printed VEGF delivery systems were recently reviewed by Poerio et al. [184]. The examples in Table 2 demonstrate the potential of 3D‐printed VEGF release systems in soft tissue engineering.

**TABLE 2:** Recent examples of 3D‐printed, hydrogel‐based scaffolds for VEGF delivery in soft tissue engineering.

Materials	Growth factor	Release profile	Application	Source
Dextran	VEGF	Rapid release in 7 days	Skin tissue regeneration	[185]
Collagen hydrogel + fibrin gel + murine neural stem cells	VEGF	—	Neural tissue regeneration	[183, 186]
PEG diacrylate + HAMA + heparin	VEGF	Controlled release	Tissue engineering/ drug delivery	[187]
GelMA	VEGF‐mimicking peptide	Slow release	Skin tissue engineering	[188]
PLA + heparin	VEGF	Sustained release in 14 days	Vascularization of implanted islets for diabetes treatment	[189]
PCL + heparin	VEGF	Sustained release for 16 days	Tissue engineering	[190]

Generally, in vitro and in vivo studies often showed benefits like better blood vessel growth in damaged tissue [183, 185, 186, 188, 191, 192, 193], however many studies struggled with accurate quantification of VEGF release, which can be influenced by multiple biological and physicochemical factors, including enzymatic degradation, local diffusion barriers, and dynamic ocular fluid turnover. Clinically, the lack of detailed studies on VEGF release raises concerns about dose control and consistent effects.

The challenge of precise VEGF dose control, which represents a limitation across hydrogels, microspheres, and 3D‐printed delivery platforms reviewed above, is directly addressable through aptamer functionalization (see Section 6 as well). The incorporation of aptamers into hydrogel matrices has been shown to reduce burst release and enable sustained, bioactive VEGF release, thereby preventing systemic toxicity [194, 195, 196, 197, 198], i.e., precisely the properties that current hydrogel systems struggle to achieve simultaneously. This enables the transformation of the hydrogel from a passive reservoir into an active, molecularly programmable delivery system. This distinction has direct implications for the potential of delivering VEGF to ocular tissues.

## 4 Administration and Delivery Strategies for VEGF‐Inhibiting Agents

The following section focuses on systems used to sequester VEGF or to deliver agents that inhibit VEGF. These systems are much more established in ocular therapies than the VEGF delivery systems (see previous section), especially for preventing or suppressing corneal vascularization.

VEGF naturally binds specific VEGF receptors (Figure 3) and heparin [88]. Therefore, the native binding principles were incorporated into carrier materials including receptors [199], affinity peptides [200], along with utilization of mAbs, fusion proteins [201, 202, 203, 204] as well as DNA‐ and RNA‐based gene therapies [205]. A few of these biological drugs are FDA‐approved, as reviewed in Formica et al. [203].

VEGF‐inhibiting agents can be delivered via multiple modalities, each with distinct administration challenges, as reviewed in the next section. We consider only administration modalities and material platforms that already use or could incorporate aptamers in future developments.

**A) Intravitreal Injections**

Intravitreal injection represents one of the most common ocular administrations of VEGF‐inhibiting drugs to treat AMD, proliferative diabetic retinopathy (PDR), diabetic macular edema (DME), or retinal vein occlusion (RVO). Generally, VEGF‐inhibiting agents could be injected in a free form in solution or associated with a carrier [206, 207]. For intravitreal injections, the following drugs were utilized: pegaptanib (aptamer) [120], bevacizumab (*Avastin*) [208], ranibizumab (*Lucentis*) (recombinant antibodies) [209], aflibercept (recombinant fusion protein combining VEGF‐receptor 1 and 2 domains and Fc‐antibody domain) [199], and many others reviewed by Xu et al. [210]. However, due to the invasive character of the treatment, side effects may occur, for example, a short‐term increase in ocular pressure, intraocular inflammation, including endophthalmitis, or the accidental injection of foreign matter like protein aggregates or silicone oil droplets, especially upon frequent application [211]. The side effect profile of repeated intravitreal injections provides a clear clinical motivation for developing sustained‐release VEGF‐sequestering platforms that reduce injection frequency.

The utilization of the aptamer pegaptanib underlines the feasibility of aptamer‐based sequestration strategies in ophthalmic environments. As further discussed in Section 6.3, aptamer‐functionalized hydrogel implants or microspheres could achieve prolonged intraocular VEGF sequestration [196] from a single administration by implementing the high affinity of VEGF‐targeting aptamers. (e.g., V7t1, *K_d_* ∼1.4 nM) [124, 125, 126] to maintain therapeutic VEGF suppression without the recurrent mechanical trauma of monthly injections.

**B) Eye Drops**

The most convenient approach to using VEGF‐inhibiting agents is to dilute the antibody in an aqueous solution (used for bevacizumab and ranibizumab). This formulation demonstrated a passive delivery to the posterior segment, and the VEGF‐inhibiting drug accumulated in the vitreous humor, the aqueous humor, and the retina [212]. In addition, the anti‐angiogenic effect of bevacizumab was compared to the activity of adiponectin, a small protein hormone with anti‐angiogenic and anti‐inflammatory properties, whereas both demonstrated equally beneficial effects in decreasing neovascularization of the cornea [213]. In addition, other small molecules that do not target VEGF directly, but inhibit its production signaling pathways, have been tested, such as a vasoinhibin analog – seven amino acid‐long cyclic peptide for angiogenic therapy in vascular retinopathies [214], a short peptide blocking VEGFR‐2 signaling for treating retinal angiogenesis [215], celecoxib/cyclodextrin micro‐suspension to inhibit cyclooxygenase‐2 [216], a cyclodextrin‐derivate including a VEGF tyrosine kinase inhibitor [217], and two synthetic N‐acetyl glucosamine derivatives interfering in the inflammatory cytokine signaling pathway [218]. Overall, mAbs and small molecules can be utilized for non‐invasive eye drop administration as a pain‐free alternative to intravitreal injections, although the bioavailability of the delivered drug is restricted due to tissue permeability and blinking (see Section 3.1). This circumstance leads to overdosage of the drug and related systemic and ocular side effects [212].

**C) Ocular Implants**

Ocular implants are devices placed in the vitreous humor and might serve as a suitable scaffold for a sustained, prolonged delivery of VEGF‐inhibiting agents. The design of an implant is challenging due to the size limitation, whereas it requires the storage of a sufficient amount of VEGF inhibitors [206]. Intravitreal implants were used to release small molecules like dexamethasone [219], axitinib (VEGF receptor inhibitor) [220], or mAbs like bevacizumab and ranibizumab [206]. Gao et al. compared dexamethasone implants and treatments including VEGF‐inhibiting agents and demonstrated comparable anatomical and functional outcomes, with differing safety profiles in retinal vein occlusion [221]. Badiee et al. reported bevacizumab‐loaded chitosan nanoparticles incorporated in a HA‐hydrogel matrix, demonstrating a prolonged, slow, sustained release over two months [222]. Furthermore, Yaylaci et al. reported an injectable ranibizumab‐loaded peptide hydrogel nanofiber system showing a controlled drug release within its therapeutic range. The implant is biodegradable, offering the possibility of injecting the implant only once, which is a clear advancement compared to recurring intravitreal injections [223]. Another strategy includes the trapping of VEGF in a bioceramic implant, based on the adsorption of VEGF, and could efficiently suppress intraocular VEGF [224].

In addition to the intravitreal implants, recently, a corneal patch was developed by Heljak et al. to prevent neovascularization. The temporary patch was composed of a GelMA hydrogel matrix and loaded with bevacizumab to prepare the eye for implantation of a corneal replacement [225]. In addition, biomaterials, such as collagen, fibrin, and agarose, are formed into engineered corneal scaffolds [226]. Such reported hydrogel‐based strategies are also highly suitable for the integration and immobilization of aptamers, offering the potential for further improvement in VEGF sequestration, as discussed in Section 6.

In general, the ocular implants are beneficial due to their long‐term usage. In posterior segment applications, intravitreal hydrogel implants provide controlled and prolonged VEGF inhibition, minimizing fluctuations in drug concentration and frequent injection. The recent expansion of hydrogel‐based VEGF‐inhibitor delivery to corneal replacement therapies introduces new possibilities for anterior segment applications, particularly in herpes‐related keratitis (HRK) and corneal neovascularization. Future clinical studies should focus on long‐term safety, efficacy, and patient‐specific considerations.

**D) VEGF‐Inhibiting Nanocarriers**

Recent progress in the development of nanosized carriers with specific characteristics that enable interference with the VEGF signaling pathways has been reviewed by Teng et al. [227]. Examples of nanocarrier systems loaded with anti‐VEGF agents are presented in Table 3.

**TABLE 3:** Recent examples of nanocarriers delivering anti‐VEGF agents.

Material of nanocarrier	VEGF‐inhibiting agent	Class	Therapy	Administration	Source
Thiolated chitosan nanoparticles	—	Antibody	RVO	Intravitreal injection	[228]
Polydopamine nanoparticles	Bevacizumab	Antibody	AMD	Intravitreal injection	[229]
PLGA/PLA nanomicelles	Ranibizumab	Antibody	AMD	Not specified	[230]
Hyperbranched mPEG‐methyl methacrylate‐co‐decyl methacrylate nanomicelles	Bevacizumab	Antibody	Posterior segment disorder	Eye drops	[231]
HA/divinyl sulfone/cholesterol nanogel	Short peptide (12 amino acids)	Peptide	AMD	Topical administration	[200]

The examples in Table 3 demonstrate the importance of VEGF‐inhibiting agent delivery for AMD treatment, which can be executed topically or via intravitreal injections. The injected materials showed a sustained release of VEGF‐inhibiting antibodies and led to reduced neovascularization [228, 229, 230]. Furthermore, the utilization of stimuli‐responsive delivery systems showed improved loading rates and prolonged release period, suggesting the potential of on‐demand release systems [229, 232]. For topical administrations, nanomicelles reveal great potential as effective delivery vehicles with increased cellular uptake and improved penetration across the corneal tissues [231].

Nanocarrier‐based delivery systems, including liposomes, polymeric nanoparticles, and micelles, enable enhanced bioavailability and prolonged drug retention, reducing the need for frequent dosing and improving therapeutic outcomes. Compared to systems that directly administrate antibody, e.g., ranibizumab or aflibercept, nanocarriers can be engineered to optimize intraocular penetration, clearance, and off‐target effects due to sustained release, thereby reducing the treatment burden and improving patient compliance, particularly in chronic conditions like AMD and DME. Future research should focus on combining nanocarrier technology with drug delivery systems, tailoring biodegradable hydrogels, stimuli‐responsive nanoparticles, and sustained‐release implants to achieve localized, controlled, and long‐lasting anti‐angiogenic effects while reducing systemic exposure and adverse effects.

Within this landscape of nanocarrier‐based delivery (see also Section 3.1D), aptamers occupy a distinctive position: unlike antibodies that require biological production under stringent conditions, aptamers are chemically synthesized (see Section 1.2) and can be conjugated to virtually any nanocarrier surface using well‐established chemistries (Section 6.1), without altering carrier morphology or drug loading capacity. The examples reviewed above, ranging from polydopamine nanoparticles to nanomicelles, demonstrate that the geometry, surface chemistry, and release profiles of nanocarriers can all be tuned independently. Aptamer functionalization could add a further dimension of tunability, enabling nanocarriers to actively bind and sequester endogenous VEGF at the target site through molecular recognition specificity.

## 5 Clinical Aspects of State‐of‐the‐Art Growth Factor Delivery

In this section, we summarize and generalize the key clinical considerations associated with ophthalmic delivery systems targeting growth factors, as described in the previous sections. Several important aspects must be taken into consideration to improve the efficacy and translational potential of the established delivery strategies: i) Although contact lenses and corneal grafts are promising carriers for the anterior segment and injectable hydrogels or particulate systems are attractive approaches for posterior segment delivery, clinically approved and experimental platforms often have poor control over drug release. This often results in an initial burst release, which may require frequent administration along with increased risk of systemic exposure through absorption via the conjunctival and nasolacrimal pathways, which can potentially disturb physiological conditions in distant organs; [233, 234, 235, 236, 237, 238, 239]. ii) In contrast to systemic drug administration, which benefits from continuous circulation and distribution, targeted drug delivery faces substantial anatomical and physiological constraints related to highly selective diffusion pathways and multiple protective barriers [14], as well as tightly regulated signaling networks (see Section 2). Together, these aspects represent significant challenges in achieving a controlled, sustained release profile that remains compatible with the eye's finely balanced physiological environment; [5, 240] iii) Additional key determinants for successful clinical translation include carrier biocompatibility, predictable and controllable degradation kinetics, minimal immunogenic or inflammatory responses, and long‐term safety under physiological conditions.

To address the above‐mentioned limitations, the implementation of aptamers – highly biocompatible, nucleic acid‐based biomolecules with tunable, specific binding capabilities (see Section 1) – represents an attractive strategy for advancing ocular therapies. We recognize their potential, particularly regarding their functional integration into biocompatible carrier systems, which would allow the binding and controlled release of exogenous GFs, or the sequestration of endogenous ones.

In this way, the clinical limitations mentioned in Sections 3 and 4, such as burst release, poor dose control, invasive administration, and inadequate retention, collectively define the performance specifications that aptamer‐functionalized platforms must address, providing a direct translational bridge between the established delivery systems and the emerging aptamer‐based strategies discussed in the following section.

## 6 Potentials and Challenges of Aptamer‐Functionalized Ocular Delivery Systems

As seen in previous sections, aptamers are underrepresented in ocular therapies, except for pegaptanib, a pegylated aptamer targeting VEGF_165_, which was the first FDA‐approved for the treatment of neovascular AMD [120]. In order to clearly delineate the opportunities, limitations and future perspectives of aptamers in drug delivery systems, we first consider different strategies for incorporating aptamers into carrier materials, as well as suitable material platforms. We then address the challenges and therapeutic potential of such aptamer‐functionalized carriers.

### 6.1 Immobilization Strategies of Aptamers

**FIGURE 7:**
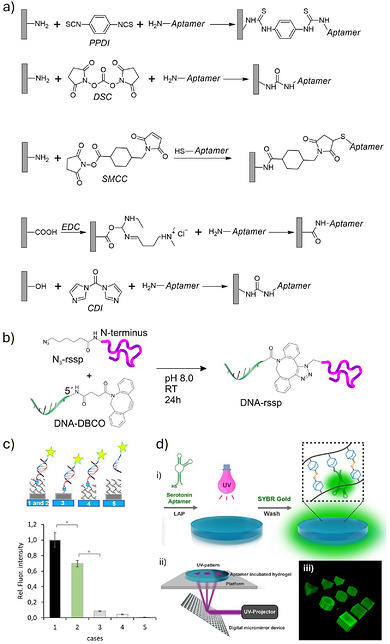
a) Common reaction sequences for covalent attachment of aptamers to surfaces (N,N'‐Disuccinimidyl carbonate (DSC), 1,4‐Phenylene diisocyanate (PPDI), carbonyldiimidazole (CDI), succinimidyl 4‐(N‐maleimidomethyl)cyclohexane‐1‐carboxylate (SMCC), 1‐ethyl‐3‐(3‐dimethylaminopropyl)carbodiimide (EDC)); b) Coupling of azide‐modified recombinant spider silk protein (N_3_‐rssp) with 5′‐dibenzocyclooctyne‐modified DNA (DNA‐DBCO) yields hybrid building blocks employed for integration of aptamers in nanohydrogel modified surfaces. Reproduced with permission from Ref. [250]. c) Upper panel: schematic representation of immobilization scenarios. (1) Nanohydrogel (nh) directly assembled from the rep‐rssp conjugate on the rssp nanofilm (nf); (2) N_3_‐rssp nh on eADF4(C16) nf functionalized by DBCO‐rep (reporter sequence) after the assembly; (3) N_3_‐eADF4(C16) nf only; (4) N_3_‐eADF4(C16) nh only; and (5) eADF4(C16) nh on eADF4(C16) nf (5). Lower panel: amounts of surface presented rep‐sequence (red strand) in the reaction setups (1–5) were compared after the hybridization of a complementary probe FAM‐cap (capture sequence, blue). Reproduced with permission from Ref. [247] d) Schematic of the aptamer patterning protocol in (i) using a thiolated aptamer and norbornene‐modified PVA gel. A chromium photomask placed between the light source and the aptamer‐loaded gel results in immobilization within illuminated locations. Aptamer presence could be visualized using DNA‐specific SYBR Gold dye. Using DLP in (ii), a UV projector directs UV light onto a digital micromirror device, which selectively projects arbitrary patterns into the gel, enabling 3D patterning of aptamers as visualized in (iii) using a z‐stack fluorescence imaging. Reproduced from Ref [253] licensed under CC BY 4.0 (creativecommons.org).

In general, immobilizing oligonucleotides on a substrate can be achieved using a variety of techniques, as has been demonstrated for biosensors and discussed elsewhere [241, 242, 243, 244]. Hereby, chemistries, such as the maleimide‐thiol Michael addition [245], the amidation of activated carboxylic groups [246] or the alkyne–azide click reaction [247] are well established, utilizing functionalized oligonucleotides and the respective functional groups exposed on the surface of interest (Figure 7a‐c). Exemplarily, using hydrogel‐based materials, hyaluronic acid could be activated with N‐hydroxysuccinimide to immobilize amino‐modified oligonucleotides [248]. The utilization of metal‐free click chemistry, such as strain‐promoted cycloaddition, enabled the covalent introduction of reactive sites within hydrogels in a bio‐orthogonal fashion. [247, 249, 250, 251] (Figure 7b,c) Further strategies for modifying hydrogel‐based materials with aptamers include free radical polymerization. For example, hydrogels for targeting VEGF delivery were formulated using a combination of acrylated PEG, gelatin, and aptamer precursors [252]. An advanced spatially defined patterning technique was developed for norbornene‐functionalized polyvinyl alcohol hydrogels using thiolated aptamers and a UV light‐sensitive photocatalyst, lithium phenyl‐2,4,6‐trimethylbenzoylphosphinate (LAP). The use of transparent hydrogels enabled the immobilization of aptamers in 2.5D using photomasking and in 3D using digital light processing (DLP) and two‐photon polymerization (2PP)‐based photolithography, respectively (Figure 7d). The approach enables precise integration of bioreceptors within biocompatible substrates [253].

Hydrogels systems are in general of interest for ophthalmic applications (see Section 3.2). Overall, high surface area and water content of hydrogels enable native‐like environments with low spatial restrictions of entrapped biomolecules [254, 255, 256], thus providing protection for sensitive biomacromolecules such as aptamers and growth factors. Another important aspect is typically low adhesiveness due to the osmotic pressure of the swollen hydrogel network, which supports antifouling and favors specific interactions in complex biological systems [247, 250]. Importantly, given the polyanionic nature of aptamers, the carrier material must be carefully selected. Non‐charged materials with low antifouling properties, such as PEG, polysaccharides (e.g., dextran), and polyanionic hyaluronan or polyacrylates, might be considered. However, polycationic matrices such as chitosan offer rather unspecific binding capacity [257], which could hinder the advantages of specific immobilization chemistries such as site specific, and hence oriented immobilization. Employing the wide range of chemistries (Figure 7), the immobilization of aptamers within hydrogel matrices is readily achievable. Hydrogels (Sections 3.2) are particularly attractive in ophthalmology due to their high water content, biocompatibility, and tissue‐like mechanical properties enabling them to conform hydrogel carries to delicate ocular tissues. However, aptamer functionalization is not limited to hydrogel systems and can be extended to alternative platforms, such as microspheres or nanoparticle‐based carriers (Sections 3 and 4), which may be tailored to specific anatomical and therapeutic requirements.

In systems with immobilized aptamers, the affinity and functionality of the aptamers after immobilization, as well as their grafting density, are crucial factors determining their therapeutic performance. We will therefore discuss these challenges in the next section.

### 6.2 Challenges in Incorporating Aptamers Into Biomaterial Platforms for Ophthalmic Applications

The chemical immobilization of DNA aptamers is associated with challenges in the accessibility and density of immobilized oligonucleotides, which is important to consider in relation to controllable loading and release of GFs: i) The immobilization density will control the dosage of GFs, which should be matched to the therapeutic window of the drug. A very high immobilization density, and thus molecular crowding, may prevent the probe from reaching the aptamer‐binding epitope [258], and may also affect the formation of proper aptamer secondary structures, which are important for target affinity; [259] ii) Restrictions in conformational freedom following immobilization should also be considered. These limitations can be addressed by introducing spacers between the aptamer and the surface, such as homologous nucleotide sequences (e.g., polyT (thymidine) or polyA (adenine)) or heterologous linkers such as PEG [260, 261, 262, 263]. Collectively, these parameters highlight that maintaining aptamer functionality after immobilization requires careful balancing of density, orientation, and surface interactions. Without such optimization, reduced affinity and impaired growth factor modulation could compromise therapeutic efficacy.

Strategies to modulate the surface density of immobilized nucleic acids are well established in biosensing applications [242]. These include controlling the density of binding sites [263, 264], the use of diluent molecules to spatially separate DNA probes [265], and adjusting the length of polyadenine or polythymidine spacer sequences between the functional sequence and the material surface [266, 267], In addition, ionic strength can be tuned to influence electrostatic repulsion of polyanionic phosphate backbone, thereby affecting probe immobilization density [268]. Such approaches are readily transferable to the functionalization of carrier materials (see previous section) intended for ophthalmic applications.

Another concerning issue is the instability of aptamers against nucleases, which represents a well‐known obstacle for nucleic acid‐based therapies [269]. In the human tear fluid, the predominant endonuclease to degrade DNA is tear lipocalin, whose endonuclease activity may play a role in protecting the ocular surface from viruses [270]. The catalytic activity of tear lipocalin is three magnitudes lower than the activity of DNase I, the predominant endonuclease in human blood [271, 272]. This comparatively low nuclease activity in the tear film supports the potential use of chemically immobilized aptamers, whose surface attachment may further reduce nuclease accessibility and thereby enhance their stability in ocular applications. Interestingly, Klose et al. further reported that DNA origami nanostructures are stable in the presence of vitreous and ocular cell media for up to seven days [273]. In contrast, RNA molecules are generally significantly less chemically stable in comparison to DNA, especially in an environment containing divalent ions. Moreover, RNA‐based aptamers may be compromised, since RNases are part of the ocular surface defense mechanism [274]. Thereby, considering stability issues for functionalization of carrier materials, DNA‐based aptamers might be more preferable [275, 276].

The nuclease‐resistance limitation can be further improved by modifying the sugar moiety, phosphodiester linkage, nucleobases, or by end‐capping of nucleic acids, as reviewed elsewhere [275, 276, 277, 278, 279]. Furthermore, the modified nucleobases and/or simulated ocular fluid might be involved in the SELEX process to evolve specifically stable aptamers [280]. The modification of nucleobases also opens up the possibility of further adjusting the affinity of aptamers toward their target [279, 281], as exemplified by the anti‐thrombin aptamer engineering [282].

The next section discusses the possibilities for the clinical translation of aptamer‐functionalized systems for the treatment of ocular pathologies.

### 6.3 Clinical Translation of Aptamer‐Functionalized Systems

In this section, we focus on the emerging opportunities for aptamer‐functionalized materials in the delivery and sequestration of GFs when applied to the anterior and posterior ocular segments. Due to the non‐covalent nature of the binding between GF and the aptamer, low‐affinity aptamers against NGF offer a potential for sustainable long‐term release (see also Section 3.1 B‐D). In contrast, aptamers with high affinity to VEGF might be used to extract the endogenous GF (Section 4).

In the anterior part of the eye, a variety of corneal degenerative, infectious, and inflammatory disorders already benefit from topical treatment using NGF. However, as described in Section 3.1A), the conventional eye‐drop administration is inefficient and costly, requiring high doses and frequent application, increasing the risk of local and systemic side effects. The use of lubricating contact lenses functionalized with NGF‐binding aptamers and loaded with rhNGF could offer several potential advantages over the current eye drops. In addition to providing mechanical protection and maintaining corneal hydration, such lenses could enable sustained and localized delivery of NGF to enhance corneal nerve regeneration and epithelial integrity. Importantly, the non‐covalent aptamer‐NGF interaction further ensures that the released protein retains its native conformation and biological activity, a critical advantage over covalent conjugation strategies that can compromise growth factor function. Furthermore, such aptamer‐functionalized contact lenses could be “recharged” by loading additional NGF. This approach may reduce the material costs, the total drug amount required as well as the frequency of administration compared with conventional topical therapy. To date, this concept has not been evaluated in clinical trials and, to our knowledge, remains unexplored in the context of NGF delivery for corneal disorders.

In addition to the aforementioned reversible corneal pathologies, corneal transplants, also known as keratoplasty, are required in cases of irreversible corneal damage resulting in vision loss. Corneal transplantations can possess a high risk for graft failure caused by inflammation, neolymphangiogenesis, and corneal neovascularization [283, 284]. Significant standard treatments to prevent graft rejection involve immunosuppressants like systemic corticosteroids and oral non‐corticosteroids, as well as VEGF‐neutralizing strategies involving anti‐VEGF antibodies (see Section 4) [107, 213]. However, strategies for the long‐term survival of corneal grafts remain underexplored, particularly in clinical and translational approaches [107]. The underrepresentation of studies regarding the anterior eye highlights the need for innovative platforms, enabling efficient VEGF sequestration in treatments of corneal neovascularization‐related pathologies. The strategies utilizing aptamer‐functionalized hydrogel‐based lenses or membranes could provide directed and localized action. In such cases, it would even be possible to release captured VEGF via affinity disruption (e.g., using a soluble form of high‐affinity aptamer or peptide ligand), and thus, the system might be renewable in harvesting capability.

Besides the anterior segment, suitable applications for aptamer‐functionalized platforms could also be envisaged for the treatment of retinal pathologies [285]. Current treatments involve surgical reattachment using scleral buckling or primary vitrectomy [286]. After the surgical reattachment, the sustainable release of NGF from correspondingly functionalized hydrogels with NGF‐targeting aptamers might support the retina healing process, since the positive effect of NGF has already been shown in experimental retinal detachment studies [287, 288]. After retinal reattachment, proliferative vitreoretinopathy (PVR), a serious postoperative complication, occurs in 5–10% of patients. Currently, no pharmacological agent for the prevention or treatment of PVR is available, making the management of PVR and retinal detachment challenging. Among the variety of involved GFs, VEGF has been evaluated as a therapeutic target, whose neutralization could prevent the development of PVR, as shown by Pennock et al. [289]. In this context, VEGF‐binding aptamer‐functionalized and injectable hydrogels or hydrogel‐microspheres could be a promising platform to sequester the growth factor onsite.

Beyond PVR, chronic conditions, such as DR – the leading cause of blindness in diabetic patients – would also benefit from VEGF‐sequestration and/or NGF‐delivering aptamer‐modified systems. Earlier stages of DR feature neurodegeneration and damage to the microvasculature of the retina. The progression of DR leading to more severe pathologies like PDR and DME is characterized by abnormalities of the vasculature and progressive microvascular damage. Standard treatment options include laser photocoagulation, vitrectomy, and VEGF‐inhibiting antibody injections, which are invasive procedures with side effects [290, 291]. Nevertheless, growth factors are heavily involved in DR, showing a decline in NGF and persistently elevated VEGF levels [290, 291, 292]. Consequently, there is a need for less invasive and potentially dual VEGF‐inhibiting and NGF‐delivering treatment options using aptamer‐modified injectable hydrogels or microspheres to reduce the frequency of injections and combine the therapeutic effects of removing VEGF and balancing NGF levels close to the retina.

## 7 Conclusion

In summary, current trends in ophthalmology reveal complementary developments in NGF delivery and VEGF sequestration, providing a strong rationale for aptamer‐based biomaterial systems. Strategies for NGF increasingly emphasize topical administration due to its accessibility and non‐invasive nature. However, high dosage requirements, limited bioavailability, and economic constraints highlight the need for more efficient and sustained delivery platforms. Emerging approaches – including NGF‐loaded contact lenses, injectable hydrogels, and particle‐based systems – reflect a clear shift toward localized, stable, and prolonged NGF modulation. By contrast, VEGF‐targeted strategies primarily emphasize inhibition rather than delivery, particularly for posterior‐segment diseases. This is due to the potent angiogenic activity of VEGF, where uncontrolled exposure carries a substantial risk of uncontrolled vascularization associated with pathological changes in ocular tissues. Current standard‐of‐care treatments are dominated by invasive intravitreal injections of monoclonal antibodies or recombinant fusion proteins, although sustained‐release implants are gaining increasing attention. Within these therapeutic paradigms, antibodies are still the dominant modality despite limitations related to cost, stability, and potential immune crosstalk. Aptamers possess several key advantages, such as robust chemical and thermal stability, adjustable binding affinity, minimal immunogenicity, and the established large‐scale synthesis following GMP standards. These properties make them attractive candidates for integration into biomaterial systems using plethora of chemistries that enable site‐specific immobilization within carriers such as injectable or 3D‐printable hydrogels, as well as micro‐ and nanoparticulate platforms. Such approaches could facilitate the development of aptamer‐functionalized implants, patches, or contact lenses capable of localized and precisely controlled GF release and/or sequestration. Avacincaptad pegol, which was approved by the FDA as an ophthalmic aptamer in 2023 alongside pegaptanib, provides dual clinical proof‐of‐concept for aptamer‐based ocular therapies, thereby strengthening the clinical translation of aptamers. Nevertheless, important challenges remain, particularly with respect to preserving aptamer functionality after immobilization and implantation. Optimizing grafting density, binding affinity, and biostability – through rational sequence engineering, chemical modifications, or the incorporation of modified nucleobases – will be critical to achieving long‐term in vivo efficacy and translation into clinics.

Overall, aptamer‐functionalized biomaterials hold significant potential to reshape future therapeutic strategies in ophthalmology by enabling controlled and precise delivery of extrinsic and sequestration intrinsic GFs. Non‐invasive anterior‐segment applications, such as NGF‐releasing contact lenses, appear particularly close to clinical translation, while sustained intraocular VEGF sequestration or VEGF delivery remains more exploratory.

## Conflicts of Interest

The authors declare no conflict of interest.

## Data Availability

The authors have nothing to report.
